# The salivary gland transcriptome of the neotropical malaria vector *Anopheles darlingi *reveals accelerated evolution of genes relevant to hematophagy

**DOI:** 10.1186/1471-2164-10-57

**Published:** 2009-01-29

**Authors:** Eric Calvo, Van M Pham, Osvaldo Marinotti, John F Andersen, José MC Ribeiro

**Affiliations:** 1 Section of Vector Biology, Laboratory of Malaria and Vector Research, National Institute of Allergy and Infectious Diseases, National Institutes of Health, Rockville, MD 20852, USA; 2 Department of Molecular Biology and Biochemistry, University of California, Irvine, CA 92697-3900, USA

## Abstract

**Background:**

Mosquito saliva, consisting of a mixture of dozens of proteins affecting vertebrate hemostasis and having sugar digestive and antimicrobial properties, helps both blood and sugar meal feeding. Culicine and anopheline mosquitoes diverged ~150 MYA, and within the anophelines, the New World species diverged from those of the Old World ~95 MYA. While the sialotranscriptome (from the Greek *sialo*, saliva) of several species of the *Cellia *subgenus of *Anopheles *has been described thoroughly, no detailed analysis of any New World anopheline has been done to date. Here we present and analyze data from a comprehensive salivary gland (SG) transcriptome of the neotropical malaria vector *Anopheles darlingi *(subgenus Nyssorhynchus).

**Results:**

A total of 2,371 clones randomly selected from an adult female *An. darlingi *SG cDNA library were sequenced and used to assemble a database that yielded 966 clusters of related sequences, 739 of which were singletons. Primer extension experiments were performed in selected clones to further extend sequence coverage, allowing for the identification of 183 protein sequences, 114 of which code for putative secreted proteins.

**Conclusion:**

Comparative analysis of sialotranscriptomes of *An. darlingi *and *An. gambiae *reveals significant divergence of salivary proteins. On average, salivary proteins are only 53% identical, while housekeeping proteins are 86% identical between the two species. Furthermore, *An. darlingi *proteins were found that match culicine but not anopheline proteins, indicating loss or rapid evolution of these proteins in the old world *Cellia *subgenus. On the other hand, several well represented salivary protein families in old world anophelines are not expressed in *An. darlingi*.

## Background

Saliva of hematophagous arthropods contain a vast array of compounds that disarm their hosts' hemostasis and inflammation, thus helping to obtain a blood meal [1,2]. In the case of mosquitoes and other blood-sucking Nematocera, saliva also helps ingestion of sugar meals, in the form of carbohydrate hydrolysing enzymes [3]. Antimicrobial products, in the form of pattern recognition proteins, serine proteases, and antimicrobial peptides (AMPs), are also routinely found in the saliva of hematophagous arthropods; these may protect the blood or sugar meal from harmful microbial growth [2].

Detailed sialotranscriptomes of several mosquito species [4-13] are revealing their salivary composition to include a number of proteins of previously known families as well as completely novel families unique to mosquitoes or their close relatives among the hematophagous Nematocera. In particular, studies done with *Culex quinquefasciatus *[8], *Aedes aegypti *[7], and *Anopheles gambiae *[13], for which the genomes are known, indicate that the mosquito salivary cocktail consists of 60–100 secreted proteins, several of which are members of multigene families. In these studies, *Aedes*-, *Anopheles*-, and *Culex*-specific proteins were discovered. Most of the salivary proteins do not have a known function but presumably affect hemostasis, inflammation, and sugar digestion or have antimicrobial activity.

Within the *Anopheles *genus, sialotranscriptomes were described for *An. gambiae *[11-13], *An. funestus *[6], and *An. stephensi *[9], all members of the same subgenus *Cellia*. These studies allowed the discovery of species-specific proteins and, importantly, that the salivary proteins among members of the same subgenus are very divergent when compared to housekeeping proteins, perhaps due to immune pressure of their vertebrate hosts, in the case of antihemostatic or antiinflammatory proteins, or of microbial resistance, in the case of antimicrobial products [9]. *An. darlingi *(subgenus *Nyssorhynchus*) is an important vector of human malaria in Central and South America, and, like all non-autogenous mosquitoes, adult females absolutely require a blood meal to develop eggs, preferring humans to other blood sources [14]. Preliminary studies with *An. darlingi *salivary glands identified one salivary lysozyme [15] and a limited proteomic work identified three additional salivary proteins [16]. Additionally, a salivary transcriptome of *An. darlingi *was previously described [5], but no protein sequences were extracted from that expressed sequence tag (EST) set. In the present work, we increased the *An. darlingi *salivary EST set from 593 to 2,371 and extracted and deposited 183 protein sequences to GenBank, 114 of which represent putative salivary secreted proteins (inclusive of alleles). This new set of proteins reveals novel proteins as well as protein families that were previously found only in *Culex*, thus pointing to their existence at 150 MYA, when a common ancestor existed to culicine and anophelines [17] and that these protein families were lost in the genus *Aedes *and the *Cellia *anopheline subgenus. Accordingly, the complex and varied evolution of salivary proteins in mosquitoes is being revealed at the same time that new protein families with potentially novel pharmacologic activities are being discovered.

## Results and discussion

### Characteristics of the assembled salivary EST set

A total of 2,371 cDNA clones were used to assemble a database [see additional file 1] that yielded 966 clusters of related sequences, 739 of which contained only one EST. This dataset included the 593 sequences used in our previous work [5]. The 966 clusters were compared, using the programs blastx, blastn, or RPS-BLAST [18], to the nonredundant (NR) protein database of the National Center of Biological Information (NCBI), National Library of Medicine, NIH, to a gene ontology database [19], to the conserved domains database of the NCBI [20], and to a custom prepared subset of the NCBI nucleotide database containing either mitochondrial or rRNA sequences.

**Figure 1:**
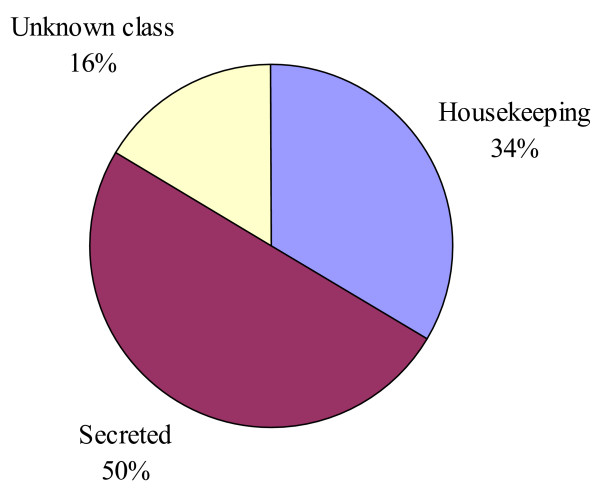
**Distribution of the transcripts from the salivary gland cDNA library of *An. darlingi *according to functional class**.

Three categories of expressed genes derived from the manual annotation of the contigs (Fig. 1). The putatively secreted (S) category contained 50% of the sequences, the housekeeping (H) category had 34, and 16% of the ESTs could not be classified and belong to the unknown (U) class. The transcripts of the U class could represent novel proteins or derive from the less conserved 3' or 5' untranslated regions of genes, as was indicated for the sialotranscriptome of *An. gambiae *[13].

### Housekeeping (H) genes

The 797 ESTs attributed to H genes expressed in the salivary glands (SGs) of *An. darlingi *were further characterized into 19 subgroups according to function (Table 1 and additional file 1). Transcripts associated with the protein synthesis machinery represented 53% of all transcripts associated with a housekeeping function, an expected result for the secretory nature of the organ. Energy metabolism accounted for 10% of the transcripts. Twenty percent of the transcripts were classified as either 'Unknown conserved' or 'Conserved secreted' proteins. These represent highly conserved proteins of unknown function, presumably associated with cellular function but still uncharacterized. These sets may help functional identification of the 'Conserved hypothetical' proteins as previously reviewed in [21].

**Table 1:** Classification of transcripts associated with housekeeping function

**Class**	**Number of transcripts**	**Percent of housekeeping group**
Protein synthesis machinery	429	53.8
Unknown conserved	114	14.3
Energy metabolism	79	9.9
Conserved secreted proteins	37	4.6
Protein modification machinery	23	2.9
Signal transduction	22	2.8
Proteasome machinery	20	2.5
Protein export machinery	15	1.9
Transcription machinery	11	1.4
Transporter/storage	10	1.3
Carbohydrate metabolism	8	1.0
Cytoskeletal	8	1.0
Nuclear regulation	6	0.8
Secondary products metabolism	6	0.8
Amino acid metabolism	4	0.5
Lipid metabolism	2	0.3
Nucleotide metabolism	1	0.1
Intermediary metabolism	1	0.1
Extracellular matrix and adhesion	1	0.1
		
**Total**	797	

### Possibly secreted (S) class of expressed genes

A total of 1,188 ESTs represent putative *An. darlingi *salivary components (Table 2 and Supplemental Table S1). These include previously known gene families as well as novel proteins. Table 2 also indicates our degree of knowledge, or ignorance, regarding these protein families, for 22 of which we have no hint for function. Many of these putatively secreted protein families of unknown function are multigenic, such as the SG1 and antigen-5 families, for example. The D7/OBP-like and aegyptin/30-kDa families contribute to 30% of all transcripts associated with secreted products. This is in line with these proteins accounting for the most intensely stained bands in SDS gels of mosquito salivary homogenates [4,7-10]. The identification of 8% of the transcripts with antimicrobial polypeptides is exceptional. Possibly this high level of expression, when compared with previous mosquito sialotranscriptomes, derives from the fact the *An. darlingi *used in this work were captured from the field and, as such, they could have been more exposed to pathogens than the laboratory-reared insects used to originate other mosquito salivary transcriptomes. Mosquito age could have been another possible variable, as the laboratory-reared mosquitoes had their glands removed in the first two days after emergence, while the ages of captured *An. darlingi *could not be specified but were most likely older than two days.

**Table 2:** Classification of transcripts associated with secreted products

**Class**		
Subclass	**Number of transcripts**	**Percent of secreted group**
**Secreted, known function**		
D7/OBP	269	22.6
Aegyptin/30-kDa antigen	98	8.2
Anophelin	20	1.7
gSG8/Kazal	14	1.2
**Immunity**		
Pattern recognition	5	0.4
Antimicrobials	92	7.7
**Enzymes**		
Glycosidases	41	3.5
Serine proteases	5	0.4
Apyrase/5' Nucleotidase	22	1.9
Peroxidase	4	0.3
**Mucins**		
gSG3 family	91	7.7
gSG10	12	1.0
13.5-kDa family	40	3.4
Other mucins	39	3.3
**Secreted, unknown function**		
SG1 family	63	5.3
SG2 family	74	6.2
SG7 family	16	1.3
SG5 family	9	0.8
Antigen-5 family	42	3.5
56-kDa family	5	0.4
Acidic protein family	32	2.7
Anopheline 6.3-kDa family	11	0.9
Anopheline 8.2-kDa family	58	4.9
Anopheline hyp 15/17 family	44	3.7
Basic tail mosquito family	32	2.7
*Culex *14.5-kDa family	2	0.2
Culicidae 23.4-kDa family	1	0.1
Culicine 41.9-kDa family	17	1.4
Other 11 families	30	2.5
		
**Total**	1188	

### The salivary secretome of *Anopheles darlingi*

From the sequenced cDNAs, a total of 183 novel *An. darlingi *protein sequences was derived, 114 of which code for putative secreted products (Table 2, Table 3, and additional file 2). Table 3 presents a summary of the secreted subset, with links to GenBank.

**Table 3:** Putative secreted proteins deducted from the salivary transcriptome analysis

**Name and link to protein sequence**	**NCBI number**	**Description**
**Secreted, known or presumed function**		

**D7/OBP protein family**		

AD-98	208657501	short form D7 salivary protein

AD-82	208657481	SHORT FORM D7 SALIVARY PROTEIN

AD-81	208657479	SHORT AD Clade D7 SALIVARY PROTEIN

AD-32	208657493	D7 short

AD-31	208657495	SHORT AD Clade D7 SALIVARY PROTEIN

AD-97	208657497	SHORT AD Clade D7 SALIVARY PROTEIN

AD-395	208657489	Short D7 protein

AD-394	208657487	Short D7 protein

AD-1	16798386	AF427696_1 D7-RELATED 3.2 PROTEIN

AD-3	16798386	D7-related 3.2 protein

AD-118	208657499	Long form D7 salivary protein

AD-560	208657485	Odorant binding protein

**30 kDa/GE rich/Aegyptin family**		

AD-24	208657597	GE rich family salivary gland protein

AD-26	208657599	30 kDa salivary antigen family protein

AD-27	208657601	GE rich salivary gland protein

AD-21	208657603	GE rich salivary gland protein

AD-22	208657605	GE rich salivary gland protein

AD-23	208657607	30 kDa salivary antigen family

AD-25	208657609	GE rich salivary gland protein

AD-28	208657617	30 kDa salivary antigen family protein

**Anophelin anti-thrombin**		

AD-99	208657573	salivary anti-thrombin peptide anophelin

AD-100	208657579	salivary anti-thrombin peptide anophelin

**gSG7/Anophensin family**		

AD-133	208657683	gSG7 salivary protein

AD-134	208657689	gSG7 salivary protein

AD-135	208657691	gSG7 salivary protein

**Kazal domain**		

AD-417	208657693	Kazal domain-containing peptide

AD-257	208657737	Kazal domain-containing peptide

AD-350	208657834	Kazal domain-containing peptide

**Mucins**		

**SG3 family**		

AD-10	208657477	SG3 PROTEIN

AD-9	208657681	sg3 protein

AD-7	208657687	sg3 protein

AD-8	208657697	SG3 PROTEIN

**gSG10 family**		

AD-143	208657645	gSG10 salivary mucin

AD-146	208657647	gSG10 salivary mucin

AD-145	208657651	gSG10 salivary mucin

**13.5 kDa family**		

AD-45	208657695	mucin-like protein

AD-46	208657701	mucin-like protein

AD-43	208657713	PUTATIVE 13.5 KDA SALIVARY PROTEIN

AD-42	208657721	putative 13.5 kDa salivary protein

AD-44	208657723	PUTATIVE 13.5 KDA SALIVARY PROTEIN

AD-41	208657733	PUTATIVE 13.5 KDA SALIVARY PROTEIN

AD-47	208657751	mucin-like protein

**Other mucins**		

AD-11	208657473	hypothetical secreted peptide precursor

AD-191	208657465	putative salivary secreted mucin 3 – fragment – similar to virus induced protein

**Peritrofins**		

AD-873	208657765	mucin-like peritrophin

**Enzymes**		

**Apyrase/5' nucleotidase**		

IS07-104	208657633	putative 5' nucleotidase/apyrase

AD-101	208657659	salivary apyrase – truncated at 5 prime

**Peroxidase**		

AD-573	208657575	salivary peroxidase

**Maltase**		

AD-70	208657611	probable salivary maltase precursor

**Serine protease**		

AD-698	208657483	CLIP-domain serine protease subfamily D – truncated at 5 prime

**Immunity related products**		

**Gambicin**		

AD-231	208657641	GAMBICIN PRECURSOR

**Defensin**		

AD-124	208657731	defensin

**Cecropin**		

AD-57	208657655	antimicrobial peptide cecropin

AD-236	208657739	antimicrobial peptide cecropin

AD-927	208657741	Cecropin precursor

**Peptidoglycan recognition protein**		

AD-457	208657711	peptidoglycan recognition protein

**Lysozyme**		

AD-174	208657469	lysozyme

AD-175	208657471	lysozyme

**Gly His rich peptide**		

AD-259	208657749	hypothetical secreted protein with GHG repeats

**Secreted, unknown function**		

**Promiscuous families**		

**Antigen 5 family**		

AD-38	33359651	Antigen 5-related 2

AD-430	208657475	antigen 5-related 2 protein

**Mosquito specific families**		

**gSG5 family**		

AD-196	208657685	conserved secreted mosquito protein

**gSG8**		

AD-178	208657639	short gSG8-like protein

**Basic tail family**		

AD-217	208657667	putative salivary secreted peptide

AD-216	208657679	putative salivary secreted peptide

**4.3 kDa family**		

AD-476	208657709	putative 4.3 kDa secreted salivary peptide

**Proline rich secreted polypeptide**		

AD-267	208657677	proline rich salivary secreted peptide

**Culicine 41.9 kDa family**		

AD-111	208657783	PUTATIVE 41.9 KDA BASIC SALIVARY PROTEIN – truncated at 5 prime

AD-112	208657807	putative 41.9 kDa basic salivary protein – truncated at 5 prime

AD-114	208657821	41 kDa family salivary secreted protein

**SG1 family**		

AD-159	208657649	SG1-like salivary protein

AD-160	208657653	SG1-like salivary protein

AD-130	208657753	GSG1 PROTEIN

AD-85	208657767	PUTATIVE SALIVARY PROTEIN SG1B

AD-86	208657777	PUTATIVE SALIVARY PROTEIN SG1

AD-153	208657781	TRIO salivary gland protein precursor – SG1 family

**gSG2 family**		

AD-49	208657761	hypothetical protein

AD-51	208657819	hypothetical secreted peptide precursor

AD-53	208657773	hypothetical secreted peptide precursor

AD-54	208657763	hypothetical secreted peptide precursor

AD-90	208657779	putative secreted peptide of the 6 kDa family

AD-91	208657785	putative secreted peptide of the 6 kDa family

AD-92	208657811	putative secreted peptide of the 6 kDa family

AD-89	208657747	putative secreted peptide of the 6 kDa family

AD-64	208657759	putative secreted peptide of the 6 kDa family

**Hyp15/17 family**		

AD-37	208657719	hypothetical salivary protein 15

AD-35	208657727	hypothetical salivary protein 15

AD-36	208657729	hypothetical salivary protein 15

**hyp8.2 kDa family**		

AD-63	208657771	hypothetical salivary protein 8.2

AD-96	208657815	hypothetical salivary protein 8.2

**hyp6.2 kDa family**		

AD-147	208657661	putative secreted salivary basic peptide hyp6.2

**hyp 5.6 kDa family**		

AD-269	208657637	hyp5.6 salivary basic secreted peptide

**2WIRRP family**		

AD-13	208657673	hypothetical secreted protein

AD-15	208657665	30 kDa salivary antigen family protein

AD-12	208657669	hypothetical secreted protein

AD-14	208657671	hypothetical secreted protein

AD-19	208657675	hypothetical secreted protein

AD-18	208657717	hypothetical secreted protein

**Other secreted peptides**		

AD-136	208657830	hypothetical conserved secreted protein

AD-138	208657848	hypothetical conserved secreted protein

AD-119	208657797	putative secreted peptide

### Proteins with presumed or experimentally validated function

#### The D7/Odorant-binding protein-like family

The first D7 protein was cloned from a cDNA library from adult female *Ae. aegypti *SGs. It had an appropriately cryptic name because, at the time, it did not match other known proteins and its function was thus unknown [22]. Additional members of this family were later described in *An. gambiae*, other mosquito species, and also in sand flies [11,23,24]. In these insects, salivary D7 proteins are encoded by multiple genes, and short and long versions of this protein family were recognized. The D7 protein family was then identified to be a member of the odorant-binding protein (OBP) superfamily [25], the long versions containing two and the short versions containing one OBP domain. Because insect OBP are known to bind and carry lipophylic compounds such as odorants and pheromones, the potential function of D7 proteins was proposed to be related to binding one or more agonists of hemostasis and thus help blood feeding [23]. This prediction was confirmed when the short D7 proteins from *An. gambiae *and the carboxy terminal domain of the long D7 of *Ae. aegypti *were found to bind biogenic amines with high affinity [26]. More recently, the amino terminal OBP domain of a D7 long form of *Ae. aegypti *was shown to bind peptidic leukotrienes with high affinity. The crystal structures of a short D7 protein from *An. gambiae *and a long D7 protein from *Ae. aegypti *revealed that the D7 OBP domains have seven alpha helices, two more than the canonical OBP family [27]. In addition to these inflammatory agonist-binding functions, a short D7 protein from *An. stephensi*, named hamadarin, was shown to inhibit bradykinin formation by inhibiting the FXII/Kallikrein pathway [28].

**Figure 2:**
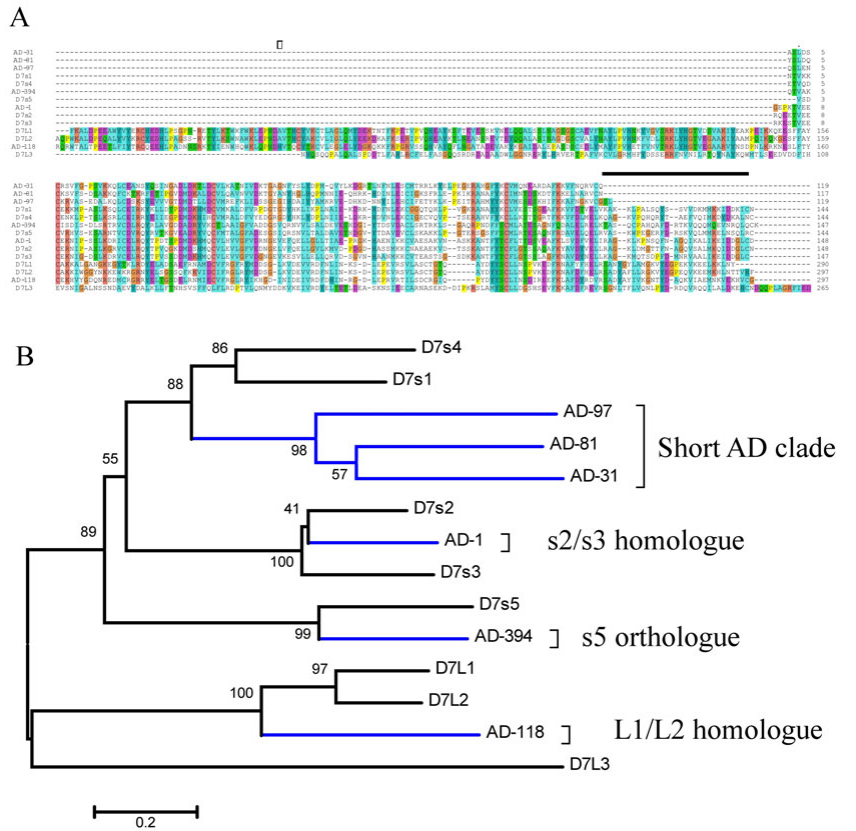
**The D7 protein family of *An. darlingi *and *An. Gambiae***. (A) Clustal alignment. (B) Phylogram based on the alignment in (A). The numbers on the tree nodes represent the percent bootstrap support in 10,000 trials. The bar at the bottom indicates 20% amino acid divergence. The *An. gambiae *sequence names start with D7 followed by s or L for short and long forms; the number following s or L represents the order of the gene in the D7 chromosomal region, following its transcription direction. The *An. darlingi *sequences start with AD, followed by a number derived from the cluster number, as determined in Supplemental Table S1. For more details, see text.

*An. gambiae *has three genes coding for long D7 proteins and five coding for the short proteins, arranged in a single contiguous gene cassette in chromosome 3R [13]. We will refer below to these proteins from *An. gambiae *by the transcriptional order that their genes appear in chromosome 3R. Twelve *An. darlingi *proteins exhibiting sequence similarity to proteins from the D7 family were identified (Table 2 and Supplemental Table S2). These include five pairs that are more than 95% identical to each other and are probably alleles. Accordingly, at least six unique products from the D7 family are identifiable in the *An. darlingi *salivary transcriptome. The alignment and phylogram of these protein sequences with all the D7 protein sequences of *An. gambiae *reveal *i*) the existence of *An. darlingi *proteins that are uniquely shorter, indicated by the bar above the alignment (Fig. 2A), which form a robust clade named 'Short AD clade' in Figure 2B. This clade is most closely related to the short D7 proteins 1 and 4 from *An. gambiae *(Fig. 2B), as indicated by strong bootstrap support; *ii*) homologues of *An. gambiae *short proteins 2 and 3 are identifiable (indicated as s2/s3 homologue in Fig. 2B), as well as the ortholog of the fifth short protein of *An. gambiae*; and *iii*) AD-118 represents an *An. darlingi *long D7 protein that is related to *An. gambiae *long D7 proteins 1 and 2.

AD-1 and AD-3, which possibly derive from a polymorphic gene, are similar to the D7s2 and D7s3 of *An. gambiae*. These proteins have in common a similar size as well as being the most transcribed D7 proteins in both species [13]. AD-1 and AD-3, but not the other *An. darlingi *D7 sequences, share an amino acid (aa) pattern, included in a cysteine framework, that are known from crystal structure to make contact with biogenic amines [27,29]. The high transcription of these gene products is in line with the large amounts of protein needed to scavenge biogenic amines that accumulate to the order of one micromolar in the host tissues [26], suggesting these *An. darlingi *proteins, likewise their *An. gambiae *homologues, function as biogenic amine scavengers.

D7s1 from *An. gambiae*, the homologue of *An. stephensi *hamadarin [28] has an alkaline pI of 9.22, contrasting with the neutral or acidic pI of the remaining short D7 proteins. To the extent this basic pI is associated with hamadarin function, it is worth noting that AD-81 and AD-31 (Fig. 2) also have pIs above 8.5, but not the more distantly related AD-97. These three *An. darlingi *proteins are members of the novel short AD clade (Fig. 2B), which shares the same tree branch where D7s1 from *An. gambiae *are located, suggesting they could have a similar function as hamadarin.

#### The 30-kDa antigen/GE-rich/aegyptin family

This protein family, found exclusively in the SGs of adult female mosquitoes, was first identified as a salivary antigen in *Ae. aegypti *[30] and later found in salivary transcriptomes and proteomes of both culicine and anopheline mosquitoes [4,6-9,13,31,32], where it was named GE-rich protein. Proteomic work also indicated that this is one of the most abundant proteins in the SGs of mosquitoes. Its gene promoter has been used to specifically drive abundant gene expression in the SGs of transgenic mosquitoes [33]. More recently, proteins of this family from *Aedes *and *Anopheles *were shown to prevent platelet aggregation by collagen [34,35], indicating conservation of function after the split of the Culicidae into the culicines and anophelines, ~150 MYA [17].

Analysis of the sialotranscriptome of *An. darlingi *allowed the identification of 8 protein sequences from this family, all represented by 2–17 ESTs found in the library. These protein sequences most probably reflect alleles from a single polymorphic gene, as they all share at least 95% identity [36]. This degree of polymorphism is paralleled in the *An. darlingi *D7 proteins but is greater than that determined in sialotranscriptomes of other mosquitoes. Possibly this high degree of sequence variability reflects our material deriving from field-caught insects, whereas previous sialotranscriptomes were made with more genetically uniform mosquito colonies.

**Figure 3:**
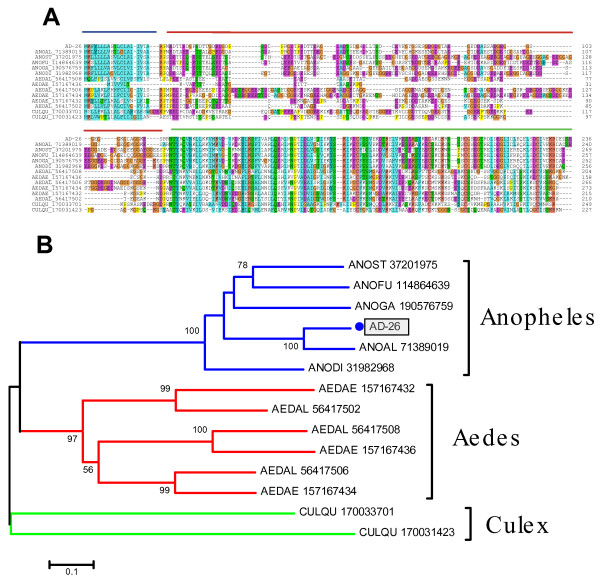
**The 30-kD/GE-rich/Aegyptin protein family of mosquitoes**. (A) Clustal alignment. (B) Phylogram based on the alignment in (A). The numbers on the tree nodes represent the percent bootstrap support in 10,000 trials (only values above 50% are shown). The bar at the bottom indicates 10% amino acid divergence. The sole *An. darlingi *sequence is identified by AD-26 and a filled circle symbol. The remaining sequences are named with the first three letters from the genus name followed by two letters from the species name and by their NCBI protein accession number. For more details, see text.

Alignment of all known members of this family, excluding those that are more than 95% identical and of the same species, shows their structure clearly to be dominated by three domains [34]: the signal secretion peptide, a Gly/Glu-rich region, and a more conserved and organized region where the block T-x(29,30)-Q-x(5)-P-x(13,15)-I-x(2)-C-F-x(20)-C-x(8,10)-C-x(19,21)-C can be identified (Fig. 3A). This block was used by the seedtop program  to search over 6 million sequences of the NR database, only retrieving mosquito proteins. The phylogram (Fig. 3B) obtained from the alignment produces strong bootstrap support for three genus-specific clades, containing three genes for *Ae. albopictus *and *Ae. aegypti*, two for *Culex quinquefasciatus*, and one for each anopheline, *An. stephensi, An. funestus, An. gambiae, An. darlingi, An. albimanus*, and *An. dirus. *The *An. darlingi *protein groups, as expected, with *An. albimanus*, another American species from the Nyssorhynchus subgenus. Near the amino terminal of the mature sequences, the Nyssorhynchus-derived 30-kDa antigen/GE-rich sequences have an RGD motif as pointed out before for the *An. albimanus *sequence [32]; this triad is not found in similar proteins of other mosquitoes. RGD-containing peptides are commonly found in snake venoms [37] and tick saliva [38], and the motif itself is usually found surrounded by two relatively close Cys groups that allow the RGD to be at the edge of a loop. This conformational feature permits the aa of the RDG motif to interact with integrins, disrupting platelet aggregation [39]. It is unknown, however, whether the RGD domain present in the 30-kDa antigen/GE-rich proteins of Nyssorhynchus mosquitoes is structurally capable of interacting with integrins.

#### Anophelin antithrombin

The salivary anticlotting agent of *An. albimanus*, named anophelin, was previously characterized as a short acidic peptide with strong thrombin inhibitory activity [40,41]. Despite extensive sequencing of the salivary transcriptomes of many hematophagous arthropods, similar sequences are found only in sialotranscriptomes of anopheline mosquitoes. Two similar *An. darlingi *cDNAs, probably corresponding to alleles of a single gene, were identified. Conceptual translation of the gene results in acidic peptides of 6.3 kDa and pI of 3.9, which are 86% identical to *An. albimanus *anophelin [42].

#### gSG7/Anophensin

The gSG7 family is also unique to anophelines. In *An. gambiae*, it has two genes coding for gSG7 and gSG72, both of which are highly enriched in female SGs [13]. More recently, the *An. stephensi *homologue was determined to inhibit kallikrein and production of bradykinin, a pain-producing substance [43]. Four putative alleles representing the homologue(s) of gSG7/Anophensin in *An. darlingi *were identified. These *An. darlingi *SG transcripts, though, have no more than 45% identity to the *An. gambiae *gSG7 and *An. stephensi *anophensin [44].

#### Kazal domain-containing peptides

The Kazal domain is ubiquitously found in proteins of metazoan organisms and, accordingly, peptides containing this domain have been identified in studies of sialotranscriptomes and proteomes of tabanids [45,46], triatomine bugs [47,48], *Culicoides sonorensis *[49], and mosquitoes [4,7,8]. In *Ae. aegypti *and *Ae. albopictus*, the transcripts encoding Kazal domain proteins were ubiquitously expressed in all major organs analyzed, suggesting their function was not specific to blood feeding [4,7]. Kazal domain peptides have also been isolated and biochemically characterized from the midgut of triatomines, where they act as anticlotting agents [50-52], and from leech saliva, where they inhibit mast cell tryptase and plasmin [53-55]. Midgut transcriptomes of sand flies have also uncovered transcription of genes encoding peptides of this class [56,57]. In addition to their classical function as protease inhibitors, Kazal domain-containing peptides were identified as the salivary vasodilator of the horse flies *Hybomitra bimaculata *and *Tabanus yao *[45,46]. In *An. darlingi*, transcripts coding for three peptides with Kazal domain were found, yielding predicted mature MW of 7.2–8.1 kDa and basic pI (8.3–9.4). AD-417 and AD-257 best match *An. gambiae *peptides when subjected to blastp against the NR database, albeit at only 45% [58] and 44% identity [59]. AD-350 best matches *Aedes *and *Culex *peptides at 47% and 51% identity [60]. The function of these salivary peptides in mosquitoes remains to be discovered.

#### Mucins and Peritrophins

Serine- and threonine-rich proteins are commonly found in sialotranscriptomes. These proteins are generally modified post translationally, and their mature forms have N-acetyl galactosamine residues, typical of mucins [61]. They probably have a function to lubricate the food canals and may also have antimicrobial function. Several protein families are represented in this group, including those previously described as SG3, gSG10, and 13.5-kDa families. Peritrophins are proteins with a chitin-binding domain that are often found in sialotranscriptomes and may be related to the maintenance of the structure of the mouthparts and/or salivary canal.

The SG3 family in *An. darlingi *is highly expressed, four proteins of which account for 90 ESTs found in the cDNA library. They may be alleles or splice variants of a single gene [62], containing 29% to 32% Ser + Thr and over 47 predicted galactosylation sites in a mature 17-kDa protein framework [63]. The *An. darlingi *SG3 has similarities only to other anopheline salivary proteins, having only 46% identity and 56% similarity to the closest relative, from *An. funestus *[64]. Compared to the Old World anophelines, the *An. darlingi *SG3 has a long GH repeat, which may confer zinc chelation capability and hence a putative antimicrobial activity for these proteins, because zinc chelation is characteristic of histidine rich antimicrobial agents that act by sequestration of this essential microbial growth factor [65-67].

The gSG10 family, containing three peptides (Supplemental Table S2), is represented by mature products with MW of 18 kDa, 22% to 23% Ser + Thr, and 15–20 predicted galactosylation sites [68]. They also may be products of a single polymorphic and/or differentially spliced gene [69]. *An. darlingi *gSG10 members match both anopheline and culicine sequences of salivary origin [70], having a unique signature block [71]that characterizes these distinctive mosquito proteins.

The 13.5-kDa protein family is also represented in *An. darlingi *by the products of two or three genes [72]. Most mosquito 13.5-kDa family members have over 30 predicted galactosylation sites [73]. *An. funestus*, *An. gambiae*, and *An. stephensi *have recognizable relatives; however those proteins show only 41% to 44% identity over most of the length of the protein to the *An. darlingi *13.5-kDa products. Culicine proteins that display only conservation of the stretch of threonine residues have been identified, but they may not be true homologues.

Two other putative mucins were found, AD-11 being a hypothetical secreted peptide of predicted mature MW of 3.8 kDa, 25% Ser + Thr, and ten potential glycosylation sites. No significant matches are found with other known proteins. AD-91, on the other hand, with 20% Ser + Thr content and 20 potential O-glycosylation sites, is 71% identical to an *An. gambiae *protein [74] that is related to a previously identified *Aedes *salivary protein and to a *Drosophila *protein annotated in the Gene Ontology database as associated with defense response to virus [75].

A single transcript in the *An. darlingi *sialotranscriptome codes for a peritrophin with a typical chitin-binding domain [76] and 69% sequence identity to an *An. gambiae *protein annotated as peritrophin A [77], which was cloned from the mosquito midgut [78].

The SG3, SG10, and 13.5-kDa families were found abundantly expressed in sialotranscriptomes of adult male *An. gambiae *[78], indicating their function is likely not related specifically to blood feeding.

#### Enzymes

Enzymes associated with both blood (apyrase and peroxidase) and sugar (amylase and maltase) feeding are known to occur in mosquito saliva; accordingly, their corresponding transcripts have been found in mosquito sialotranscriptomes. Serine protease-encoding transcripts also are regularly found, but their proposed functions in helping blood feeding by interacting with host proteins or as participants in immune proteolytic cascades have not been validated.

Apyrase, which hydrolyses ATP and ADP to AMP and orthophosphates, has been a ubiquitous finding in the saliva of blood-sucking arthropods, where it destroys these important agonists of inflammation and platelet aggregation [2,79]. Mosquitoes have co-opted the 5' nucleotidase family to achieve this function [80-82]. Two genes of this family are expressed in the SGs of *An. gambiae *[13], named putative 5' nucleotidase and salivary apyrase, although both may function redundantly as apyrases. The sialotranscriptome of *An. darlingi *presents evidence for the two orthologues, IS07-44, a full-length orthologue of the salivary 5' nucleotidase of *An. gambiae *[83], to which it is 66% identical, and AD-101, which is a 5' truncated clone best matching the *An. gambiae *salivary apyrase [84].

A peroxidase was previously identified as the vasodilator for norepinephrine-induced aortic contractions found in *An. albimanus *SGs [85,86]. AD-573 encodes the full-length sequence of an *An. darlingi *salivary peroxidase that is 86% identical to *An. albimanus *and 52% identical to *An. gambiae *salivary peroxidases [87]. This type of salivary vasodilator is so far unique to anopheline mosquitoes.

Maltase and amylases, as well as their transcripts, have been regularly found in the saliva and sialotranscriptomes of mosquitoes [88-91]. The first cloned gene from the SGs of any mosquito was actually a member of this family [92]. *Ae. aegypti *and *An. gambiae *express both genes in their SGs. Transcripts coding for both enzymes were found in the sialotranscriptome of *An. darlingi *[see additional file 1]. The full-length sequence for the orthologue of *An. gambiae *salivary maltase (68% identity) [93] is presented in Supplemental Table S2.

Transcripts coding for at least two different serine proteases were found in *An. darlingi *sialotranscriptome [see additional file 1]. Supplemental Table S2 presents a truncated sequence of a CLIP domain serine protease expressed in *An. darlingi *SGs, 86% identical to the *An. gambiae *closest match [94].

#### Immunity-related products

Antimicrobial peptides, lysozyme, and pathogen pattern recognition polypeptides are commonly found in the sialotranscriptome of blood-sucking arthropods. Among the AMPs found in the sialotranscriptome of *An. darlingi*, a gambicin [95], a defensin [96], and three different cecropins [97] are described in their full-length condition. A peptidoglycan recognition protein, 94% identical to an *An. gambiae *protein [98], is also reported as a full-length protein. Additionally, this study [see additional file 1] provides evidence for *An. darlingi *transcripts coding for C-type lectins and ficolins, and an odd transcript having a full PMEI Pfam domain [99] normally found in plant proteins associated with inhibition of microbial pathogens' pectin methyl esterase. Two similar lysozyme cDNAs, probably products of alleles, are also described as full-length polypeptides, matching 57% identity to the closest *An. gambiae *protein [100]. Another identified lysozyme, contig 443 [101], corresponds to a previously described salivary *An. darlingi *lysozyme [15]. The occurrence of multiple lysozymes in the *An. darlingi *sialome is not surprising, as two lysozymes are expressed in the *An. gambiae *SGs [13].

With less certainty, we include in the immunity-related products the full-length sequence for a Gly-His-rich peptide that might have antimicrobial function by zinc chelation, as explained above. This protein matches a *C. quinquefasciatus *salivary peptide that also contains Gly repeats and a poly His in the amino terminus [102].

### Secreted proteins with unknown function

#### Promiscuous antigen 5 (AG5) family

This is a ubiquitous protein family found in animals and plants [103] and in all sialotranscriptomes of blood-sucking Diptera analyzed so far. The function of these proteins in mosquito saliva is not known, although they were implicated in a proteolytic function in the venom of the marine snail *Conus textile *[104], in toxic functions in the saliva of a venomous lizard and snake venoms [105-109], and in an antifungal function in plants [110]. Remarkably, a member of this family acquired a typical RGD domain surrounded by Cys residues and acts as a main platelet aggregation inhibitor in the horsefly *Tabanus yao *[46]. Several genes from the AG5 family are transcribed in the SGs of mosquitoes, including some specific to the adult females and thus possibly associated with a specific function in blood feeding [4,7,13]. We present evidence, in the form of full-length transcripts, for the expression of at least two members of the AG5 family in *An. darlingi *SGs [111]. AD-38 matches with 67% identity the putative gVAG protein precursor of *An. gambiae *[112], a transcript enriched in the adult female SGs when compared with expression in other tissues [13]. AD-430 matches *An. gambiae *AG5-related 2 protein [113], which was shown to be ubiquitously expressed in adult female tissues [13]. The function(s) of this protein family in mosquitoes remain to be determined.

#### Mosquito-specific gSG5 family

Transcripts coding for the gSG5 protein [114] were first discovered in the SGs of *An. gambiae *and shown to be exclusively expressed in the adult female SGs [13,115]. This protein produces weak similarity to a salivary protein of *Ae. aegypt*i [116] and better similarity to other *Aedes *[117] and *Culex *proteins [118], indicating this is a mosquito-specific protein. Six transcripts coding for this protein were found in the sialotranscriptome of *An. darlingi*. AD-196 is 46% identical to the *An. gambiae *orthologue and only 26% and 23% identical to the culicine proteins [119]. The function of this mosquito-specific protein remains unknown, but its tissue- and sex-specific expression profile suggests it is possibly related to blood feeding.

#### Mosquito-specific gSG8 family

The gSG8 is a highly divergent family, with members only from *An. gambiae *and *Ae. aegypti *[120]. Alignment of the three sequences displays a conserved motif L-C-W-A-x-K-x(2)-P-T-A-x(6)-C-x(5)-K, which might help identify new members of this family. In *An. gambiae*, this protein is specifically expressed in female SGs [115], suggesting a likely role in blood feeding.

#### Mosquito-specific basic tail family

**Figure 4:**
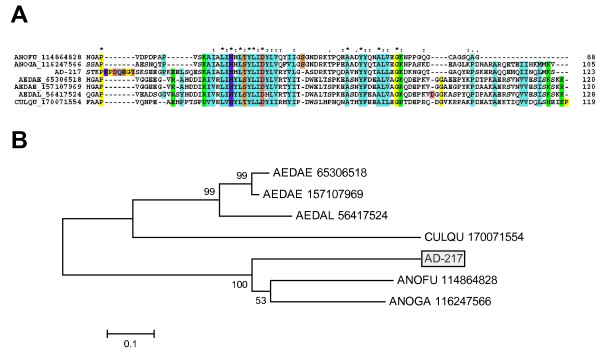
**The salivary basic tail family of mosquito proteins**. (A) Clustal alignment. The sole *An. darlingi *sequence is identified by AD-217. The remaining sequences are named with the first three letters from the genus name followed by two letters from the species name and by their NCBI protein accession number. Conserved cysteines are shown in black, hydrophobic conserved amino acids (aa) in light blue, conserved Pro and Gly in yellow, conserved bulky non-charged aa (Asn, Gln, Ser, Thr) in grey, conserved Ser + Thr in brown, conserved negatively charged aa in red, identical positively charged aa in violet, conserved charged aa in green. The symbols above the alignment indicate: (*) identical sites; (:) conserved sites; (.) less conserved sites. (B) Phylogram derived from the alignment in (A). The numbers on the tree nodes represent the percent bootstrap support in 10,000 trials (only values above 50% are shown). The bar at the bottom indicates 10% aa divergence.

AD-216 and AD-217 represents two similar proteins deducted from two and three ESTs, respectively. They may represent splicing variants or alleles of the same gene [121]. The predicted mature peptides have 11.2 kDa and solely match proteins found in other mosquito sialotranscriptomes or other hypothetical mosquito proteins [122]. The basic tail name derives from a conserved Lys-X-X-Lys or Lys-X-X-Arg found in the carboxyterminus of proteins derived from the genus *Aedes *but lacking in the anopheline sequences. The alignment indicates a conserved backbone and the absence of cysteine residues, from where the block pattern L-x-H-x-L-x-Y-L-x-D-x(17,18)-A-x(2)-Y-x(3)-A-x(3)-G can be deduced (Fig. 4A). The derived phylogram (Fig. 4B) follows the expected mosquito phylogeny. *Ae. aegypti *transcripts coding for the basic tail peptide were enriched in adult female SGs [7].

#### Mosquito-specific 4.3-kDa family

**Figure 5:**
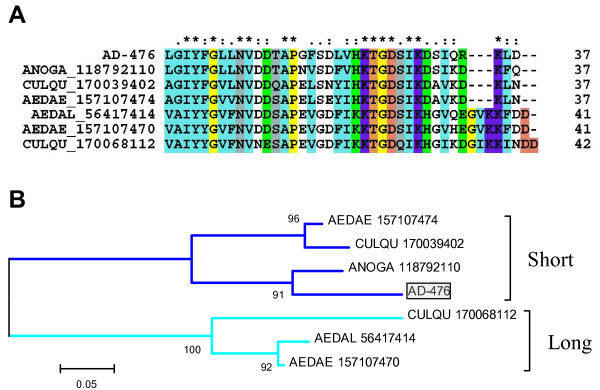
**The salivary 4.3-kDa family of mosquito proteins**. (A) Clustal alignment. The sole *An. darlingi *sequence is identified by AD-476. The remaining sequences are named with the first three letters from the genus name followed by two letters from the species name and by their NCBI protein accession number. Conserved cysteines are shown in black, hydrophobic conserved amino acids (aa) in light blue, conserved Pro and Gly in yellow, conserved bulky non-charged aa (Asn, Gln, Ser, Thr) in grey, conserved Ser + Thr in brown, identical negatively charged aa in red, identical positively charged aa in violet, conserved charged aa in green. The symbols above the alignment indicate: (*) identical sites; (:) conserved sites; (.) less conserved sites. (B) Phylogram derived from the alignment in (A). The numbers on the tree nodes represent the percent bootstrap support in 10,000 trials (only values above 50% are shown). The bar at the bottom indicates 5% aa divergence.

AD-476 represents the peptide sequence of a mature protein of 4.1 kDa having significant similarities only to other polypeptides found previously in culicine mosquito sialotranscriptomes or predicted proteomes of mosquitoes [123]. This is the first time a protein of this family is found in an anopheline sialotranscriptome. Alignment and phylogram of the mature predicted peptides shows that *Ae. aegypti *and *C. quinquefasciatus *have two such peptides, those of *Anopheles *matching the slightly smaller version (Fig. 5A). The derived phylogram indicates two clades grouping the short and the large forms. In *Ae. aegypti*, transcripts coding for a member of this family were shown to be enriched in the adult female SGs [7].

#### Culicine proline-rich secreted protein

The sialotranscriptome of *Ae. aegypti *identified a protein named proline-rich salivary secreted peptide [124], close homologues of which were never found in other sialotranscriptomes. Transcripts for this protein were found exclusively on the adult female SGs of *Ae. aegypti*, indicating a function related to acquisition of the blood meal [7]. The sialotranscriptome of *An. darlingi *provided three ESTs, which when assembled derive the sequence AD-267, matching this *Aedes *protein at 47% identity [125] and also, weakly, a smaller region of a salivary protein from *An. stephensi *of the same size. AD-267 was subjected to psiblast analysis against the NR database retrievieng only sequences from *Ae. aegypti*, which converged after two iterations. The presence of AD-267 in *An. darlingi*, its homology to the *Ae. aegypti *protein, and its absence in *An. gambiae *suggest that the gene for this family existed in the ancestor of culicines and anophelines but was lost or modified beyond recognition in *Culex *and the *Cellia *subgenus of *Anopheles*.

#### Culicine 41.9-kDa family

**Figure 6:**
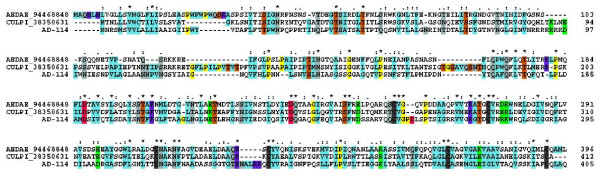
**Clustal alignment of the 41.9-kDa family of mosquito proteins**. The sole *An. darlingi *sequence is identified by AD-114. The remaining sequences are named with the first three letters from the genus name followed by two letters from the species name and by their NCBI protein accession number. For more details, see text. Conserved cysteines are shown in black, hydrophobic conserved amino acids (aa) in light blue, conserved Pro and Gly in yellow, conserved bulky non-charged aa (Asn, Gln, Ser, Thr) in grey, conserved Ser + Thr in brown, conserved negatively charged aa in red, identical positively charged aa in violet, conserved charged aa in green. The symbols above the alignment indicate: (*) identical sites; (:) conserved sites; (.) less conserved sites.

**Figure 7:**
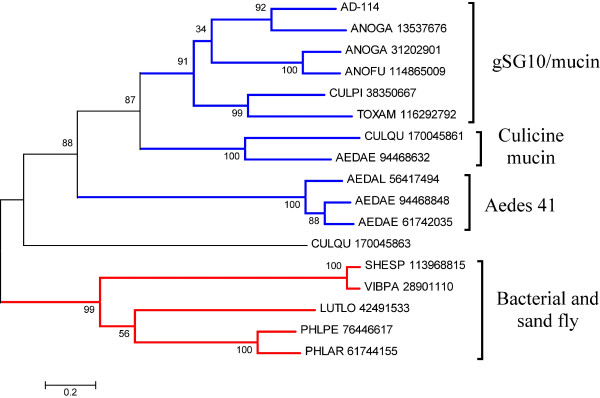
**The expanded 41.9-kDa family**. Phylogram based on the alignment of sequences derived from the use of the PSI-BLAST tool to retrieve sequences on the NR database from the NCBI using as seed the *An. darlingi *sequence AD-114. The numbers on the tree nodes represent the percent bootstrap support in 10,000 trials (only values above 50% are shown). The bar at the bottom indicates 20% amino acid divergence. Except for the *An. darlingi *sequence, the remaining sequences are named with the first three letters from the genus name followed by two letters from the species name and by their NCBI protein accession number. For more details, see text.

The first 41.9-kDa family member was characterized in sialotranscriptome of *Ae. aegypti *and later found in *C. quinquefasciatus *and in *Ae. albopictus *[4,7,8,10]. It has never been found in any anopheline sialotranscriptome, nor does it have any similar protein predicted from the *An. gambiae *genome [126]. AD-114, however, produces similarities to 41.9-kDa family members when subjected to blastp analysis against the NR database [127]. The blast results interestingly retrieves other salivary proteins from hematophagous Diptera from the NR database, such as gSG10, gSG9, and other mucins, despite having itself only three potential galactosylation sites. The alignment of the *An. darlingi *protein with the 41.9-kDa proteins from *Ae. aegypti *and *C. quinquefasciatus *shows extensive similarities over the whole length of the sequences, including a conserved cysteine framework, despite having less than 30% identity with the culicine proteins (Fig. 6). AD-114 thus appear to be a "missing link" joining previously thought unrelated salivary protein families from Culicines and Anophelines. To further investigate this possibility, we used psiblast to search AD-114 against the NR database, retrieving mostly proteins found before in sialotranscriptomes of blood-sucking Diptera [128], including Culicoides [49] and sand flies [129,130]. In addition to the known 41.9-kDa members from culicines, the anopheline proteins annotated as gSG10 and gSG9 are also retrieved, as are a group of proteins annotated as salivary mucins from mosquitoes, including the non-bloodfeeding species *Toxorhynchites amboinensis *[131]. Exceptionally, two bacterial proteins are retrieved, as well as one from the wasp *Nasonia vitripennis*. The alignment of the proteins from Diptera plus the two bacterial proteins by the Clustal tool does not reveal any region of common conservation among all proteins (not shown), but the derived bootstrapped phylogram (Fig. 7) is informative. Strong support is obtained for four clades, as indicated in Figure 7. The first clade includes sequences from both anopheline and culicine mosquitoes annotated as gSG10, gSG9, and mucins, together with the *An. darlingi *sequence. A second clade includes *Culex *and *Aedes *proteins annotated as mucins. This second clade roots with strong bootstrap support to the previous clade. A third clade includes *Aedes *proteins annotated as 41-kDa protein, or a short version, annotated as 30.3-kDa protein. This clade also roots strongly with the two previous clades. The sole *C. quinquefasciatus *sequence shown in Figure 7 (gi|170045863), the 41.9-kDa basic salivary protein, does not group significantly with any other sequence. Finally, a fourth clade groups together the bacterial and sand fly proteins. This clade does not root with strong bootstrap support to the previous clades. The presence of the bacterial proteins in this clade is puzzling, and suggests that the Nematocera proteins could have derived from bacterial contaminants. However, the proteins deriving from *Ae. aegypti*, *C. quinquefasciatus *and *An. gambiae *map to assembled chromosomes or supercontigs, and their respective genes contain introns indicating they are of eukaryotic origin. Together, these results support the argument that the 41.9-kDa protein family of mosquitoes has a common salivary ancestor before the split of anophelines and culicines, being recognized in *An. darlingi *by AD-114; in the *Cellia *subgenus, the 41.9-kDa protein family has evolved to produce shorter proteins, the subfamily members of the gSG10 and gSG9 families. Sand flies express related salivary proteins that might have been acquired by convergent evolution or share a distant common ancestor that can no longer be recognized with the available sequences.

#### Anopheline-specific SG1 family

**Figure 8:**
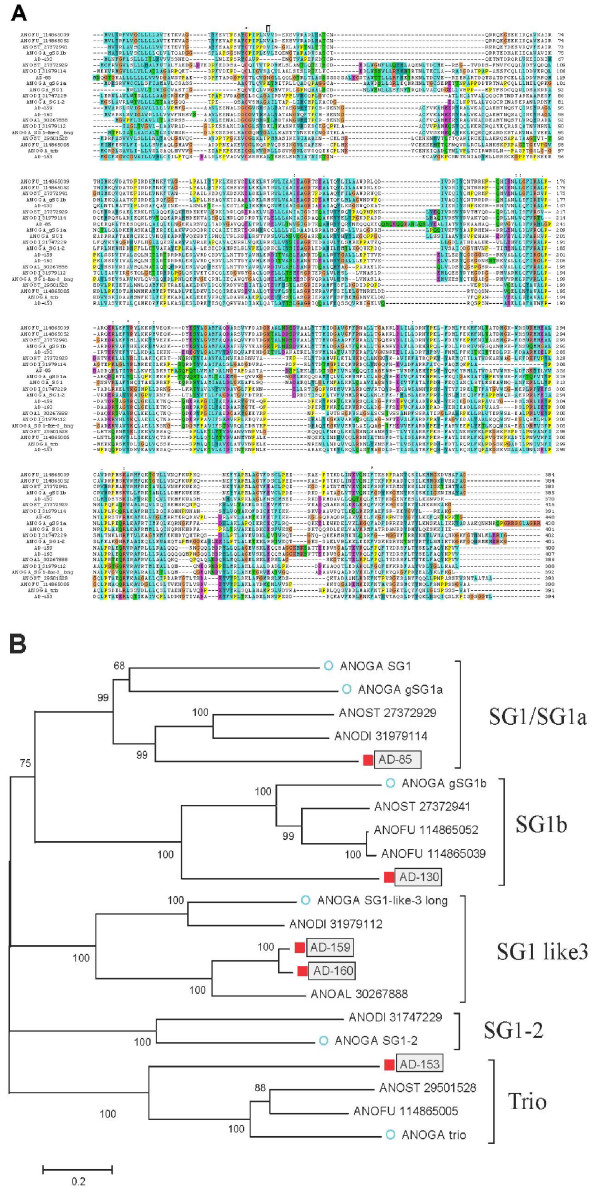
**The G1 protein family of anopheline mosquitoes**. A) Clustal alignment. (B) Phylogram based on the alignment in (A). The numbers on the tree nodes represent the percent bootstrap support in 10,000 trials (only values above 50% are shown). The bar at the bottom indicates 20% amino acid divergence. The *An. darlingi *sequences are identified by AD and a filled square symbol. The *An. gambiae *sequences are identified by a circle and are named as reported before [7]. The remaining sequences are named with the first three letters from the genus name followed by two letters from the species name and by their NCBI protein accession number. For more details, see text.

Six genes coding for proteins of this unique protein family were found in *An. gambiae *salivary transcriptomes [11,12,115], four of which are located as a contiguous gene cluster [132] in chromosome X [13]. Remarkably, all these genes are uniexonic, unusual for eukaryotic genes coding for these relatively large proteins, attaining a mature molecular weight above 40 kDa, suggesting its acquisition as horizontal transfer. This gene family appears to be specifically associated with SG function. The transcripts coding for the Trio, SG1, and SG1b proteins appears to be exclusively expressed in the female SGs, while SG1-like3 and gSG1-2 and gSG1a are enriched in the female glands but also present in lower amounts in male glands and not observed in other tissues [13]. When these proteins were subjected to blastp against the NR database, only other anopheline sequences are retrieved. Sixty-three ESTs were found in the *An. darlingi *sialotranscriptome coding for proteins of this family, from which six full-length clones were sequenced. Of these six sequences, two possibly derive from alleles or splice variants [133]. When full-length protein sequences from all known members of this family are aligned by the Clustal tool, very few conserved aa are identified (Fig. 8A); however, the deduced phylogram show strong bootstrap support for five clades (Fig. 8B), named for the *An. gambiae *proteins, as follows: Clade SG1/SG1a contains these two proteins from *An. gambiae *and also one sequence each from *An. stephensi*, *An. dirus*, and *An. darlingi*. Clade SG1-like3 contains two sequences from *An. darlingi *that could be the result of a recent gene duplication or polymorphism and splice variation [134]. These two sequences cluster with strong bootstrap support, as expected, with the sole sequence from *An. albimanus. *The Trio clade also has AD-153 from *An. darlingi. *The clade SG1-2 is the only clade not having *An. darlingi *representatives. The function of these proteins remains to be determined.

#### Anopheline-specific SG2 family

The SG2 protein was deduced from salivary *An. gambiae *cDNAs and shown to be expressed in female glands and adult males but not in other tissues [11]. It derives from a single gene in chromosome 2L and is abundantly transcribed in sialotranscriptomes of male *An. gambiae *[135]. Related, but very divergent, sequences were obtained solely from salivary transcriptomes of other anopheline species [6]. The sialotranscriptome of *An. darlingi *indicates that at least two different genes exist coding for proteins of this family. One gene codes for mature proteins of 8.5 kDa, from which four alleles or splice variants are derived [136]. A second gene may have produced another five different alleles or splice variants coding for shorter (5.6- to 6.1-kDa) peptides [137], but it is more likely that these derive from two closely related genes. Comparison of these proteins with other anopheline sequences displays sequence identities varying from only 26% [138]to 31% [139]. Because this protein family is expressed in both male and female *An. gambiae *[11,135], and due to its relatively small size, it may display antimicrobial function.

#### Anopheline-specific hyp 15/hyp 17 family

The hyp 15 and hyp 17 proteins, previously identified in sialotranscriptomes of *An. gambiae *[12], have alkaline pI and ~4.7 kDa. Their genes reside as tandem repeat in chromosome X and are preferentially expressed in adult female SGs [13]. Homologues were additionally found in *An. stephensi *and *An. funestus. *The *An. darlingi *sialotranscriptome presents evidence of three transcripts that may derive from splice variants from a single gene [140], which are 41% and 39% identical to the *An. funestus *and *An. gambiae *homologue [141].

#### Anopheline-specific hyp 8.2/hyp 6.2 family

In *An. gambiae*, the genes coding for the hyp 8.2 and hyp 6.2 proteins are found as a tandem repeat in chromosome arm 2L. These proteins have mature molecular weight of 6–9 kDa, do not have sequence similarity, and are grouped together solely by virtue of being chromosomal neighbours. Transcripts coding for these two polypeptides are similarly enriched in *An. gambiae *adult female SGs [13]. *An. stephensi *and *An. funestus *also have members of these protein families. In *An. darlingi*, two quite divergent protein sequences [142] deduced from the sialotranscriptome are similar to hyp 8.2 [143], and one is similar to hyp 6.2 [144].

#### Anopheline-specific hyp 5.6 family

*An. darlingi *protein AD-269 has a predicted molecular weight of 6.5 kDa and matches [145] the carboxyterminus of a salivary peptide named hyp 5.6 previously described in *An. gambiae *sialotranscriptome [13]. Members of this family have not been found previously in other sialomes. In *An. gambiae *the transcript coding for hyp 5.6 was ubiquitously transcribed, suggesting a housekeeping or antimicrobial role.

#### Anopheles 2WIRRP salivary hypothetical protein

**Figure 9:**
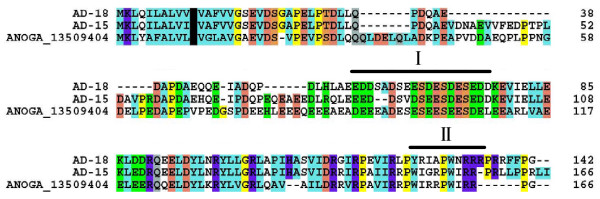
**The 2WIRRP family of Anopheline proteins**. Clustal alignment of the *An. darlingi *proteins with the *An. gambiae *homologue. Background colour follows convention as in Figure 6. Bar labelled I indicates region of Ser [Asp/Glu] [Asp-Glu] repeats. Bar labelled II identifies the WIRRP repeats notable on the *An. gambiae *sequence.

A protein cryptically named hypothetical protein was previously identified in a cDNA library of *An. gambiae *[115], but homologues were never found in other sialotranscriptomes of either anopheline or culicine mosquitoes. This *An. gambiae *protein produces matches to other unrelated sequences in the NR database by virtue of repeated acidic amino acids. The sialotranscriptome of *An. darlingi *produced 60 transcripts matching this *An. gambiae *protein, distributed into six putative protein sequences deriving from possibly two genes [146], of which AD-18 represents a shorter form of the family (Fig. 9). The five remaining deduced sequences may result from alleles [147]. These proteins have predicted mature molecular weight of 14–17 kDa and pI of 4.2. They are 41% [148] to 50% identical [149] to the *An. gambiae *homologue. Alignment of two of the *An. darlingi *sequences with the *An. gambiae *homologue identifies a region of Ser [Asp/Glu] [Asp-Glu] repeats (identified with a bar labelled I in Fig. 9) and a region of two repeats WIRRP in the *An. gambiae *sequence (identified with a bar labelled II in Fig. 9), which provides a name for the family.

#### *An. darlingi *salivary-secreted orphan proteins

Two *An. darlingi *protein sequences, never before evidenced in mosquito sialotranscriptomes, are described here with clear signal peptide indicative of a secretion. These are AD-136, which significantly matches only hypothetical proteins of *An. gambiae, Ae. aegypti*, and *C. quinquefasciatus *[150], and AD-119, which has no significant matches to any known protein in the NR database. Seven and 15 transcripts were found coding for each protein, respectively. AD-136 has an allele [151], AD-138, derived from two transcripts.

#### *An. darlingi *salivary absentee proteins

In a previous sialotranscriptome analysis of *An. gambiae*, 92 transcripts from a total of 4,066 [13] coded for a protein named gSG6 [115], orthologues of which were found in *An. stephensi *and *An. funestus *sialotranscriptomes [152]. Considering that we have sequenced in the present work 2,371 ESTs from *An. darlingi*, some 53 ESTs would have been expected for this protein. None were found, suggesting this family to be specific for the *Cellia *subgenus. Similarly, the related *An. gambiae *proteins named hyp 10 and hyp 12 [153] had 37 and 12 corresponding ESTs, but none were found in the *An. darlingi *cDNA library, also suggesting this family to be *Cellia*-specific.

### Comparison of protein sequence identities between *An. darlingi *and *An. gambiae *gene products

Seventy-seven deduced protein sequences coding for putative housekeeping (H) products are presented in Supplemental Table S2. These proteins allow comparison of the evolutionary rate of the S proteins compared with that of the H proteins, using the *An. gambiae *proteome as a reference set as done before for comparing *An. stephensi *salivary proteins with those of *An. gambiae *[9]. For this comparison, we used only protein sequences from *An. darlingi *that had at least 100 aa of alignment to an *An. gambiae *protein, as identified by blastp with the filter for low complexity set to off. The protein identity in the two groups, 86% for the H and 53% for the S group, were significantly different (*P *< 0.001, Mann-Whitney rank sum test) (Table 4), supporting the concept that the evolution of mosquito salivary-secreted proteins occurs at a faster pace than housekeeping proteins.

**Table 4:** Identity at amino-acid level between *Anopheles darlingi *and *An. gambiae *salivary secreted and housekeeping proteins

**Secreted protein name**	**Name**	**Length**	**% identity**
AD-32	Short D7r4	137	35
AD-97	Short D7r4	130	29
AD-395	Short D7r5	156	53
AD-1	Short D7r3	169	61
AD-118	Long D7 1	309	43
AD-23	30-kDa antigen	252	59
AD-133	gSG7 anophensin	134	47
AD-8	SG3 mucin	139	34
AD-143	gSG10	188	59
AD-47	13.5-kDa mucin	149	34
AD-104	Apyrase	571	66
AD-573	Peroxidase	592	86
AD-38	gVAG	261	67
AD-430	Antigen 5	254	51
AD-196	gSG5	328	46
AD-217	Basic tail	116	48
AD-159	SG1-like3	376	33
AD-130	gSG1b	351	35
AD-86	SG1	409	30
AD-153	Trio	383	29
AD-138	Unknown secreted	241	60
AD-191	Virus-induced mucin	277	71
AD-457	Peptidoglycan recognition protein	188	94
AD-174	Lysozyme	138	80
AD-70	Maltase	567	80
			
Mean		272.6	53.2
SE		29.0	3.8
SD		144.9	19.1
			

**Housekeeping protein name**	**Name**	**Length**	**% identity**
AD-519	Tetraspanin	249	85
AD-680	Unknown conserved	188	90
AD-408	Tetraspanin	288	74
AD-184	Unknown conserved	144	86
AD-527	Unknown conserved	101	87
AD-77	Unknown conserved	137	45
AD-79	Unknown conserved	137	45
AD-401	Unknown conserved	270	56
AD-584	Ferritin	231	65
AD-94	Conserved secreted protein	104	84
AD-345	Conserved secreted protein	126	92
AD-640	Conserved secreted protein	126	90
AD-189	Conserved secreted protein	119	79
AD-939	Conserved secreted protein	136	30
AD-870	N-methyl-D-aspartate receptor-associated protein	100	87
AD-489	Phosphatidic acid phosphatase	298	66
AD-205	40S ribosomal protein SA (P40)/Laminin receptor 1	290	82
AD-195	Ribosomal protein S4	262	91
AD-220	60S ribosomal protein L7	258	84
AD-398	Similar to 3-hydroxybutyrate dehydrogenase type 2	255	90
AD-165	60S ribosomal protein L7A – truncated at 5 prime	253	80
AD-224	60s ribosomal protein L2/L8	252	96
AD-167	40S ribosomal protein S3A	247	93
AD-295	emp24/gp25L/p24 family of membrane trafficking protein	211	92
AD-328	Peptidyl-prolyl cis-trans isomerase	202	89
AD-207	Ribosomal protein L19	190	95
AD-225	60S ribosomal protein L9	190	88
AD-201	60s ribosomal protein L18	189	86
AD-193	60S ribosomal protein L11	188	93
AD-222	60S ribosomal protein L22	187	95
AD-710	Nucleoside diphosphate kinase	168	93
AD-246	60S ribosomal protein L21	162	83
AD-126	40S ribosomal protein S19	157	88
AD-156	Ribosomal protein L22	154	84
AD-180	60S ribosomal protein L13A	154	79
AD-212	40S ribosomal protein S11	153	92
AD-215	60s ribosomal protein L24	153	89
AD-251	40S ribosomal protein S14	152	99
AD-253	60S ribosomal protein L26	151	94
AD-937	Hypothetical conserved protein	150	92
AD-235	40S ribosomal protein S15	149	94
AD-151	Ribosomal protein S16	146	96
AD-241	60S ribosomal protein L14/L17/L23	140	100
AD-252	40S ribosomal protein S12	136	97
AD-281	H3 histone, family 3A	136	99
AD-245	Ribosomal protein L32	134	93
AD-592	Mitochondrial ribosomal protein L54	134	83
AD-230	40S ribosomal protein S17	131	98
AD-239	Ubiquitin-like/40S ribosomal S30 protein fusion	131	76
AD-240	40S ribosomal protein S15/S22	130	97
AD-229	Ubiquitin/60s ribosomal protein L40 fusion	128	100
AD-242	Ribosomal protein S8	126	84
AD-280	H2A histone family, member V	126	95
AD-250	60S ribosomal protein L31	124	99
AD-185	40S ribosomal protein S20	120	92
AD-117	Acidic ribosomal protein P1	115	89
AD-247	60S ribosomal protein L36	113	95
AD-120	60S acidic ribosomal protein P2	113	82
AD-262	Mitochondrial F1F0-ATP synthase, subunit Cf6	107	90
AD-116	Translation elongation factor EF-1 alpha/Tu	103	97
			
Mean		167.1	86.1
SE		7.1	1.8
SD		55.0	13.8

## Conclusion

Anophelines diverged from culicine mosquitoes approximately 150 MYA [17]. Within anophelines, the new world species diverged from the old world forms concomitantly or before the breakup of Gondwanaland, at ~95 MYA [154]. Within the anophelines, detailed sialotranscriptome analyses have been made only from members of the *Cellia *subgenus (*An. gambiae*, *An. stephensi*, and *An. funestus*). In addition, detailed sialotranscriptomes and proteome data are available for three culicines, *Ae. aegypti*, *Ae. albopictus*, and *C. quinquefasciatus*, and one mosquito of the subfamily Toxorhynchitinae, *T. amboinensis*. The insertion of a neotropical anopheline (subgenus Nyssorhyncus) fills a gap of information and helps to explain mosquito evolution with regard to adaptation to blood feeding through their salivary proteins.

From a conservative perspective, the sialotranscriptome of *An. darlingi *confirms the presence of ubiquitous salivary mosquito protein families, such as the D7, 30-kDa antigen/aegyptin, mucins, AG5, gSG5, gSG8, basic tail, the enzymes apyrase/5' nucleotidase and amylase/maltase, and the immunity-related proteins lysozyme, defensin, cecropin, and Gly-His-rich peptides; most of these proteins are uniquely found in mosquitoes. From another standpoint, the *An. darlingi *sialotranscriptome has confirmed the presence of proteins so far known exclusively in anopheline mosquitoes, such as the antithrombin anophelin, the SG1, SG2, hyp 15/hyp 17, hyp 8.2/hyp 6.2, hyp 5.6, 2WIRRP. In the last two cases, the 2WIRRP and hyp 5.6, the *An. darlingi *sequences represent the second member of the family previously discovered in *An. gambiae *but never before found in other anophelines.

Of interest, the *An. darlingi *sialotranscriptome also produced protein sequences with similarity to polypeptides previously found exclusively in culicine sialotranscriptomes, such as the proline-rich secreted protein, Kazal domain-containing peptides, and the 41.9-kDa family. Psiblast analysis of the *An. darlingi *sequence member of the 41.9-kDa family allowed identification of related *Cellia *anopheline sequences members previously known as gSG10 and gSG9, indicating these two families may have evolved quite rapidly from 41.9-kDa ancestors that are now absent not only in the *An. gambiae *known sialotranscriptome, but also from any predicted protein from this mosquito genome (Fig. 7). On the other hand, *An. darlingi *lacks transcripts coding for proteins abundantly transcribed in *An. gambiae *and other *Cellia *mosquitoes, indicating the loss – or evolution beyond recognition – of these protein families in *An. darlingi *evolution.

Finally, the rapid divergence of salivary proteins allows the possibility of using such *An. darlingi *proteins as specific markers of vector exposure, as is now being attempted for *An. gambiae *and *Ae. aegypti *[155-158]. Additionally, to the extent that the rapid divergence of the salivary proteins is not associated with divergence of function, the differences between orthologous salivary proteins between *An. gambiae *and *An darlingi*, and also among anophelines of the different subfamilies, represents a natural site-directed mutagenesis experiment that will help identify structural determinants of function in such bioactive proteins [159-161].

## Methods

### Mosquitoes and cDNA library construction

The sequences utilized in this study originated from the same cDNA library used in our previous publication [5]. This cDNA library was derived from SGs dissected from adult female *An. darlingi *of unknown ages that were field caught in Porto Velho, Rondonia, Brazil. PolyA^+ ^RNA was extracted from 60 dissected pairs of SGs using the Micro-FastTrack mRNA isolation kit (Invitrogen), which was then used to make a PCR-based cDNA library using the SMART™ cDNA library construction kit (BD Biosciences-Clontech) as described before [10].

### cDNA sequencing

The SG cDNA library was plated on LB/MgSO_4 _plates containing X gal/IPTG to an average of 250 plaques per 150-mm Petri plate. Recombinant (white) plaques were randomly selected and transferred to 96-well Microtest™ U-bottom plates (BD BioSciences) containing 100 μl of SM buffer (0.1 M NaCl; 0.01 M MgSO_4_; 7 H_2_O; 0.035 M Tris HCl [pH 7.5]; 0.01% gelatin) per well. The plates were covered and placed on a gyrating shaker for 30 min at room temperature. The phage suspension was either immediately used for PCR or stored at 4°C for future use.

To amplify the cDNA using a PCR reaction, 4 μl of the phage sample was used as a template. The primers were sequences from the λ TriplEx2 vector and named pTEx2 5seq (5' TCC GAG ATC TGG ACG AGC 3') and pTEx2 3LD (5' ATA CGA CTC ACT ATA GGG CGA ATT GGC 3'), positioned at the 5' end and the 3' end of the cDNA insert, respectively. The reaction was carried out in 96-well flexible PCR plates (Applied Biosystems) using FastStart Taq polymerase (Roche) on a GeneAmp^® ^PCR system 9700 (Perkin Elmer Corp.). The PCR conditions were: one hold of 95°C for 3 min; 25 cycles of 95°C for 1 min, 61°C for 30 sec; 72°C for 5 min. The amplified products were analysed on a 1.5% agarose/EtBr gel. cDNA library clones were PCR amplified, and those showing a single band were selected for sequencing. Approximately 200–250 ng of each PCR product was transferred to ThermoFast 96-well PCR plates (ABgene Corp.) and frozen at -20°C before cycle sequencing using an ABI3730XL machine. The obtained sequences were submitted to DBEST and have the GenBank accession numbers FK703778–FK705605.

### Primer extension experiments on selected clones

These were performed using sequencing primers designed by the Primer3 program [162], aimed at a region ~100 bp upstream (5') of the end of the previously obtained sequence information of high quality. The process was repeated until full length information was obtained. The primer extension sequences were submitted to DBEST and have the accession numbers FL688077–FL688134. The sequences representing the open reading frames shown in supplemental table 2 have been deposited to GenBank and have the accession numbers EU934251–EU934432.

### Bioinformatic tools and procedures

ESTs were trimmed of primer and vector sequences. The BLAST suite of programs [18], CAP3 assembler [163] and ClustalW [164] software were used to compare, assemble, and align sequences, respectively. Phylogenetic analysis and statistical neighbour-joining (NJ) bootstrap tests of the phylogenies were done with the Mega package [165]. For functional annotation of the transcripts we used blastx [18] to compare the nucleotide sequences with the NR protein database of the NCBI and to the Gene Ontology (GO) database [19]. The program reverse position-specific BLAST (RPS-BLAST) [18] was used to search for conserved protein domains in the Pfam [166], SMART [167], Kog [168], and conserved domains databases (CDD) [20]. We have also compared the transcripts with other subsets of mitochondrial and rRNA nucleotide sequences downloaded from NCBI and to several organism proteomes downloaded from NCBI, ENSEMBL, or VectorBase. Segments of the three-frame translations of the EST (because the libraries were unidirectional, six-frame translations were not used) starting with a methionine found in the first 300 predicted aa, or the predicted protein translation in the case of complete coding sequences, were submitted to the SignalP server [169] to help identify translation products that could be secreted. O-glycosylation sites on the proteins were predicted with the program NetOGlyc [170]. Functional annotation of the transcripts was based on all the comparisons above. Following inspection of all these results, transcripts were classified as either secretory (S), housekeeping (H) or of unknown (U) function, with further subdivisions based on function and/or protein families.

## Abbreviations

aa: amino acid; AMP: antimicrobial peptide; AG5: antigen 5 family; EST: expressed sequence tag; H class: housekeeping; NR: nonredundant; OBP: odorant-binding protein; S class: secreted; SG: salivary gland; SMART: switching mechanism at 5' end of RNA transcript; U class: unknown function.

## Authors' contributions

EC and JFA helped with library manufacture, sequencing, data analysis, and contributed to the manuscript. VMP participated in sequencing the NIH library. OM helped with experiment design and contributed to manuscript. JMCR performed data analysis and contributed to the manuscript. All authors read and approved the final manuscript.

## Supplementary Material

Additional file 1 **Assembled and annotated sialotranscriptome of *An. darlingi *female mosquitoes.** Hyperlinked Excel spreadsheet and associated files with EST assembly results. This is a compressed ZIP file that should be expanded to a new directory. After this is done, start Excel and then open the file ending in .xls so the hyperlinks will work. Click here for file

Additional file 2 **Annotated sialotranscriptome of *An. darlingi *female mosquitoes.** Hyperlinked Excel spreadsheet with deducted protein sequences. See description above. Click here for file
